# The relation between surgical approaches for pelvic ring and acetabular fractures and postoperative complications: a systematic review

**DOI:** 10.1007/s00068-022-02118-3

**Published:** 2022-11-25

**Authors:** Robert A. Timmer, Cassidy Q. B. Mostert, Pieta Krijnen, Sven A. G. Meylaerts, Inger B. Schipper

**Affiliations:** 1 Leiden University Medical Center, Leiden, The Netherlands; 2 Haaglanden Medical Centre, The Hague, The Netherlands

**Keywords:** Pelvic ring fractures, Acetabular fractures, Surgical approaches, Post-operative complications, Risk factors

## Abstract

**Introduction:**

Although many articles report complications after pelvic ring and acetabular fracture surgery, a general overview of complication rates and potential risk factors is lacking. The current review provides a comprehensive summary of the complications after pelvic ring and acetabular fracture surgery in relation to the surgical approach.

**Material and Methods:**

Pubmed and Embase databases were systematically searched using the key words: pelvic fracture, acetabular fracture, fixation, surgical approaches, complications, and their synonyms. Extracted data included patient and fracture characteristics, surgical approaches, and post-operative complications; surgical site infections (SSI), implant-related complications, malunion and non-union. Study data were summarized using descriptive statistics.

**Results:**

Twenty-two studies (twenty-one retrospective cohort studies, of which three comparative, and one randomized controlled trial) were included in this review. The overall complication rates reported for the included surgical approaches were: 17% for the (Modified) Stoppa approach, 11% for percutaneous fixation, 5% for the Kocher–Langenbeck approach, 7% for the ilioinguinal approach and 31% for external fixation. The most frequent complications were SSI (22%) and neurological (31%) complications, which were most often reported in patients treated with an external fixator. Re-operation rates were comparable for the surgical approaches (4–8%). Two studies reported on risk factors and identified concomitant traumatic injuries, prolonged ICU stay and high body mass index as risk factors for SSI.

**Conclusion:**

External fixation of pelvic fractures is associated with highest complications rates including SSI’s and neurological complications. Although post-operative complications are frequently reported after pelvic fracture surgery, more studies are needed that identify potential risk factors. These will assist the surgeon in (pre)operative decision making and development of preventive strategies.

**Supplementary Information:**

The online version contains supplementary material available at 10.1007/s00068-022-02118-3.

## Introduction

Pelvic fractures including pelvic ring and acetabular fractures, represent a broad spectrum of injuries. Minor pelvic ring or acetabular fractures are usually the result of low energy trauma, while major pelvic ring fractures mainly result from high-energy trauma (HET) and are diagnosed in up to 25% of young severely injured patients [1–3]. HET-related pelvic fractures are especially associated with high mortality rates ranging between 20 and 50% [1–9]. In extensive or displaced acetabular fractures and in unstable pelvic ring fractures, fixation is often required to restore stability and joint congruity for acceptable long-term functional results. In patients with signs of hemodynamic instability caused by major pelvic ring and/or acetabular fractures, acute temporary stabilisation followed by a secondary definite fixation may be needed to obtain acute haemorrhage control and to prevent exsanguination [10].

Although novel and less invasive operation techniques are emerging, open reduction and internal fixation (ORIF) remains the gold standard for those cases that cannot be percutaneously fixated, providing optimal fracture exposure and achieving the best long-term results for both acetabular and pelvic ring fractures [11]. While there are many different surgical approaches to perform ORIF, selecting the appropriate surgical approach for obtaining optimal fracture exposure is fundamental in the management of these types of fractures. In general, every surgical intervention may be associated with post-operative complications. The extent of the approach for fracture fixation of the pelvis may vary depending on the type and location of the fracture, as well as on other patient-related factors. Due to differences in anatomical location, the extent of the dissection and duration of the operation, different surgical approaches for both acetabular and pelvic ring fracture fixation pose varying risks of post-operative complications [12, 13]. These complications, including surgical site infections, may lead to impaired wound healing, hardware removal, and eventually to poor long-term functional outcomes.

Although many articles have addressed complications after pelvic ring and acetabular fracture surgery, a general overview summarizing post-operative complications per surgical approach and their potential risk factors is lacking. This systematic review aims to present a comprehensive overview of these complications in relation to specific surgical approaches for pelvic ring and acetabular fracture fixation.

## Material and methods

This systematic review was conducted according to the PRISMA (Preferred Reporting Items for Systematic reviews and Meta-Analyses) guidelines [14]. A literature search was conducted on 12–02-2022 in the online databases Pubmed and Embase, using a search strategy composed in close collaboration with a trained medical librarian (Appendix I). Title and abstract of the identified articles were screened using the following selection criteria: (1) adult patients (aged ≥ 18 years), (2) patients with operatively treated pelvic or acetabular fractures, (3) studies reporting on surgical complications including but not exclusively wound complications, implant-related complications, neurological complications, (4) study size ≥ 20 patients, and (5) published in English, Dutch or German.

Case reports, studies published before 2000, studies concerning pathological fractures, primary prosthesis surgery or studies reporting on surgical approaches other than the (modified) Stoppa, minimally invasive anterior plate osteosynthesis (MIPO), ilioinguinal, Kocher–Langenbeck, percutaneous approach, pararectus, or external fixator placement, were excluded. The full text of the studies meeting the inclusion criteria was read and selected if meeting the same selection criteria. Studies reporting on multiple approaches were excluded if complications were not reported per approach. Study selection and data extraction were conducted independently by two authors (RT, CM).

### Data extraction

The following data were extracted from the included studies: study design, patient characteristics, trauma mechanism, fracture classification, surgical approaches, post-operative complications, and re-operations (including secondary placement of a total hip prosthesis).

Post-operative complications included surgical site infections (SSI), implant-related complications (defined as plate and/or screw breakage and/or complaints related to osteosynthesis material), malunion (healing of the bone in an abnormal position), non-union (failure of the fractured bone to heal) and neurological complications.

Differentiation between deep and superficial infections was considered but not performed since the included articles provided insufficient information or used heterogeneous definitions. Implant-related complications, neurological complications and mal- and non-union were scored if the included studies described these as a surgery-related complication.

Data were divided into subgroups based on fracture type (pelvic ring or acetabulum) and surgical approach.

### Assessment of risk of bias

Risk of bias was independently assessed by the two reviewers (RT, CM) using the methodological index for non-randomized studies (MINORS) criteria [15]. For non-comparative studies, this tool includes eight methodological aspects that are scored as 0 (not reported), 1 (reported but inadequate) or 2 (reported and adequate), with a maximum score of 16. For comparative studies, the tool includes 4 additional criteria (maximum score 24) [15].

### Data analysis

Study data were reported by fracture type and surgical approach using descriptive statistics (number with percentage, mean with standard deviation or median with range). Complication rates were calculated and displayed as percentages of the included patients across the studies per surgical approach.

## Results

**Fig. 1:**
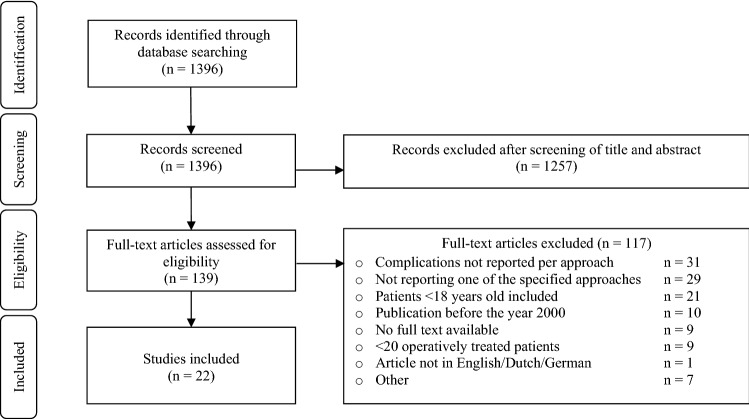
Flowchart of article selection

The literature search identified 1396 potentially relevant articles. After screening titles and abstracts, 139 studies were selected for full text screening. After careful reading of the full text articles, twenty-two studies with a total of 1395 patients met the inclusion criteria and were included in this review (Fig. 1) [16–37]. Twenty-one studies had a retrospective study design of which three were comparative cohort studies [18, 24, 26]. One randomized control trial (RCT) [21] was included. Thirteen studies comprising 702 patients reported on post-operative complications after pelvic ring fracture surgery (Table 1) [16–28]. Nine studies with a total of 693 patients reported on the post-operative complications after acetabulum fracture surgery (Table 2) [29–37]. Follow-up periods ranged between 6 and 68 months. The study of Iqbal et al. presented complications of two surgical approaches in acetabular fractures: the ilioinguinal approach and the Kocher–Langenbeck approach [34]. According to the MINORS criteria the methodological quality of the included studies was poor to moderate (Table 3).

**Table 1:** Characteristics of studies on pelvic ring fractures per approach

												Complications			
Study	*N*^a^	Age^b^		Male %	Fracture type (%)	HET/LET^c^	Operative approach	Type of fixation	FU^d^	Overall %	SSI %	Implant related %	Neurological %	Non-union %	Re-operation %
**(Modified) Stoppa approach**															
Bastian et al. [16] 2016, Germany, Retrospective	63		53 (16–88)	73	Tile B2: 11(17) Tile B3: 14 (22) Tile C1: 22 (35) Tile C2: 3 (5) Tile C3: 13 (21)	NR	Modified Stoppa	Reconstruction plate	3.3 years (1.0–7.9)	13	5	8	2	5	10
Oh et al. [17] 2016, Korea, Retrospective	22		41 (23–61)	45	LC: 12 (54) APC: 4 (18) VS: 6 (27)	High energy	Stoppa	Plate	16 months (10–51)	0	0	0	0	NR	NR
**Percutaneous approach**															
Chen et al. [18] 2012, China, Retrospective	*Group A: 29*		Group A: 37 (11)	*Group A: 69*	Tile C1: 19 (33) Tile C2: 8 (14) Tile C3: 2 (3)	NR	*ORIF*^a^	*Group A: reconstruction plate*	*23.81* *Days* (± 6.18)	*0*	*0*	*0*	*0*	NR	*0*
	Group B: 29		Group B: 39 (10)	Group B: 72	Tile C1: 21 (36) Tile C2: 6 (11) Tile C3: 2 (3)	NR	Percutaneous	Group B: sacroiliac screws	23.94 days (± 6.03)	7	0	0	7	NR	7
Dekimpe et al. [19] 2020, France, Retrospective	32		46 (24–80)	NR	Tile B: 7 (22) Tile C: 23 (78)	NR	Percutaneous	Sacroiliac and acetabular screws	13.5 months (4–30)	9	0	9	NR	NR	6
Falzarano et al. [20] 2018, Italy, Retrospective	96		37 (19–63)		Tile C: 96	High energy	Percutaneous	Sacroiliac screws	NR (1 – 60 months)	12	12	0	NR	0	2
Li et al. [21] 2014, China, RCT	Group A: 32 *Group B: 32*		Group A: 39 (22–57) *Group B: 37.3* *(21–55)*	Group A: 78 *Group B:75*	Tile C1: 22 (35) Tile C2: 8 (12) Tile C3: 2 (3) Tile C1: 23 (36) Tile C2: 8 (12) Tile C3: 1 (2)	High energy High energy	Percutaneous	Group A: Sacro iliac screws *Group B:* *Control, Sacro iliac Anterior plate fixation*	NR NR	3 *9*	3 *9*	0 *0*	0 *0*	NR NR	3 NR
Osterhoff et al. [22] 2011, Switzerland, Retrospective	25		56 (20)		LC I and LC II or Tile B2	NR	Percutaneous	Sacroiliac screws	6 months (6–4)	16	0	8	NR	8	24
Rommens et al. [23] 2020, Germany, Retrospective	128		Median 69 (51–81)	33	FFP 1: 1 (1) FFP 2: 38 (30) FFP 3 : 11 (9) FFP 4: 15 (12) Tile B: 36 (128) Tile C:27 (21)	High energy 63 Low energy 65	Percutaneous	Transpubic screws	27.6 weeks (7.0–73.5 weeks)	12	2	7	0	6	8
Wenning et al. [24] 2021, Germany, Retrospective Comparative	Group A: 48 *Group B: 29*		Group A: 79 (14) *Group B: 62 (18)*	Group A: 17 *Group B: 41*	Tile C0: 22 (29) Tile C2: 26 (34) Tile C3: 0 Tile C0: 15 (19) Tile C2: 0 Tile C3: 14 (18)	NR NR	Percutaneous *Open*	Group A: Sacroiliac screws *Group B:Lumbo pelvic fixation*	At least 6 months At least 6 months	13 *18*	2 *14*	11 *4*	NR *NR*	0 *4*	13 *18*

*SSI* surgical site infection; *LC* lateral compression; *APC* anterior–posterior compression; *VS* vertical shear; *FFP* fragility fracture of the pelvis; *NR* not reported

^a^Number of included patients

^b^Median + range or mean + SD when no range is reported

^c^HET = High energy trauma, LET = low energy trauma

^d^Reported as mean ± standard deviation or as median (IQR)

**Table 2:** Characteristics of studies on acetabular fractures per approach

											Complications			
Study	*N*^a^	Age^b^	Male %	Fracture type (%)	HET/LET^c^	Operative approach	Type of fixation	FU^d^	Overall %	SSI %	Implant related %	Neurological %	Non-union %	Re-operation %
**(Modified) Stoppa approach**														
Khoury et al. [29] 2012, Israel, Retrospective	60	NR	NR	NR	NR	Modified Stoppa	Plate and/or screws	NR	25	5	NR	0	NR	NR
Isaacson et al. [30] 2014, USA, Retrospective	36	47 (16)	86	AC: 1 (3) Trans: 5 (14) T-type: 6 (17) TPW: 2 (6) ACPH: 7 (19) BC: 15 (42)	High Energy	Modified Stoppa	Plate and/or screws	32 months (9–59)	17	8	NR	6	6	8
Singh et al. [31]2020, India, Retrospective	30	40 (18–60)	80	AC: 3 (10) T-type: 1 (3) TPW: 1 (3) ACPH: 6 (20) BC: 19 (63)	High energy	Modified Stoppa	Plate and/or screws	6 months (NR)	17	3	NR	6	NR	NR
Verbeek et al. [32] 2018, Netherlands, Retrospective	45	51 (17)	71	AW: 3 (7) Trans: 8 (18) T-type: 16 (36) ACPH: 5 (11) BC: 13 (29)	High energy 69% Low energy 31%	Modified Stoppa	Plate and/or screws	59 months (12–165)	9	4	2	NR	NR	8
**Kocher-Langenbeck approach**														
Alexa et al. [33] 2013, Romania, Retrospective	42	41 (26–71)	69	PW: 9 (21) PC: 3 (7) PCW: 4 (10) Trans: 11 (26) T-type: 5 (12) TPW: 10 (24)	High energy	KL	Plate and (non-specified) screws: 39 (non-specified) Screws only: 3	NR (1–4 years)	9	2	NR	7	NR	5
Iqbal et al. [34]^e^ 2017, Pakistan, Retrospective	170	45 (22)	59	NR	NR	KL	Reconstruction plate	At least 12 months (NR)	5	3	NR	NR	0	NR
Negrin et al. [35] 2010, Austria, Retrospective	27	A: 40 (10) B: 42 (20)	41	Trans: 27	NR	KL	Plate osteosynthesis	9 months (± 6)	12	0	0	12	NR	15
Suzuki et al. [36] 2009, USA, Retrospective	326	43 (18)	77	PW: 78 (23.9) PC: 7 (2.1) AW: 1 (0.5) AC: 22 (6.7) PCW: 16 (4.9) Trans: 29 (8.9) T-type: 35 (10.7) TPW: 48 (14.7) ACPH: 21 (6.4) BC: 69 (21.2)	NR	KL	Plate and/or screws	NR	5	5	NR	NR	0	5
Kumar et al. [37] 2021, India, Retrospective	80	43 (13)	60	PW: 27 (34) PC: 16 (20) PCW: 9 (11) Trans: 7 (9) T-type: 9 (11) TPW: 7 (9) ACPH: 5 (6)	High Energy	KL	Plate and/or screws	mean 2.6 years (SD)	3	1	NR	2	NR	NR
**Ilioinguinal approach**														
Iqbal et al.[34]^e^ 2017, Pakistan, Retrospective	45	45 (22)	59	NR	NR	Ilioinguinal	Reconstruction plate	At least 12 months (NR)	7	7	NR	NR	NR	NR

*SSI* surgical site infection; *KL* Kocher-Langenbeck; *AC* anterior collum; *AW* anterior wall; *ACPH* anterior column posterior hemitransverse; *BC* both columns; *PW* posterior wall; *PC* posterior column; *PCW* posterior column + wall; *Trans* transverse type; *T-Type* T-shape type; *TPW* transverse posterior wall; *NR* not reported

^a^Number of included patients

^b^Median + ( range) or or mean + (SD)

^c^HET = High energy trauma, LET = low energy trauma

^d^Reported as mean ± standard deviation or as median (IQR)

^**e**^This study is mentioned twice; once for the Kocher-Langenbeck approach and once for the ilioinguinal approach. The total study population consisted of 261

**Table 3:** Methodological quality assessment according to the MINORS criteria (0: not reported; 1: reported but inadequate; 2: reported and adequate)

Study	Aim of the study	Inclusion of consecutive patients	Prospective collection of data	Endpoint appropriate to the study aim	Unbiased evaluation of endpoints	F/U period appropriate to the major endpoint	Loss to F/U not exceeding 5%	Sample size calculation	Additional criteria				
									Adequate control group	Contemporary groups	Baseline equivalence of groups	Adequate statistical analyses	Total score
Bastian et al. [16]	2	1	0	2	0	2	2	1	NA	NA	NA	NA	10/16
Oh et al. [17]	2	2	0	2	0	2	1	1	NA	NA	NA	NA	10/16
Chen et al. [18]	2	2	0	2	0	2	2	1	0	2	2	1	16/24
Dekimpe et al. [19]	2	1	0	2	0	2	2	1	NA	NA	NA	NA	10/16
Falzarano et al. [20]	1	0	0	2	0	2	0	0	NA	NA	NA	NA	5/16
Li et al. [21]	2	2	2	2	1	2	2	1	2	2	2	1	21/24
Osterhoff et al. [22]	2	1	0	2	0	1	0	1	NA	NA	NA	NA	7/16
Rommens et al. [23]	2	1	0	2	0	1	0	0	NA	NA	NA	NA	6/16
Wenning et al. [24]	2	2	2	2	0	2	1	0	1	1	1	2	16/24
Scaglione et al. [25]	1	1	0	2	0	0	0	0	NA	NA	NA	NA	4/16

*NA* not applicable

### Post-operative complications per fracture type

Post-operative complications after surgery for pelvic ring fractures were reported in 0–59% of patients, for acetabular fractures this range was 3–25% of patients. Most post-operative complications concerned SSI, varying from 0 to 35% in patients with pelvic ring fractures [16–28] and from 0 to 8% in patients with acetabular fractures [29–37], depending on the type of fracture fixation and surgical approach (Tables 1 and 2).

Implant-related complications such as screw malposition, plate breakage after pelvic ring surgery were reported in eleven studies ranging from 0 to 23% of patients [16–24, 26, 27]. Two studies reported implanted-related complication percentages after acetabular fracture surgery of 0% and 2% of patients [32, 35]. Post-operative neurological complications after pelvic ring surgery were reported in nine studies and ranged from 0 to 10% of patients [16–18, 21, 23, 25–28]; six studies on acetabular fracture surgery reported a range from 3 to 12% of patients [29–31, 33, 35, 37].

Non-union was reported in seven pelvic ring studies with percentages ranging from 0 to 8% of patients [16, 20, 22–26]. Three acetabular fracture studies reported non-union rates from 0 to 6% of patients [30, 34, 36]. Re-operation rates varied between 0 and 24% in patients with pelvic ring [16, 18–25, 27] and between 5 and 15% in patients with acetabular fractures [30, 32, 33, 35, 36].

### Post-operative complications per surgical approach

#### (Modified) Stoppa approach

The overall complication rate for the (modified) Stoppa approach was 17.3%. SSI was the most frequent complication, occurring in 5.5% of patients with fractures of the pelvic ring or acetabulum (Table 4).

**Table 4:** Post-operative complications per surgical approach

	Overall complications, %	SSI, %	Implant related, %	Neurological, %	Non-union, %	Re-operations, %
(Modified) Stoppa approach	17.3	5.5	4.6	2.5	7.2	5.2
Percutaneous (Screw)	11.0	4.4	4.4	1.1	3.3	7.5
External fixator	31.1	22.1	12.9	31.0	0	4.3
Kocher Langebeck approach	5.4	3.1	0	5.2	0	6.2
Illioinguinal approach	7.0	7.0	NR	NR	NR	NR

*SSI* surgical site infections; *NR* not reported

Two studies described the results of in total 85 patients undergoing pelvic ring fracture surgery via a (modified) Stoppa approach [16, 17]. In both studies all included patients underwent plate osteosynthesis. Surgical site infections occurred in 0% and 5% of patients, implant related complications in 0–8% (Table 1).

Bastian et al. reported reoperations in 10% of the included patients. Three patients needed a surgical debridement because of deep infection, one patient suffered from post-operative hematoma for which surgical evacuation was required, one patient had an abdominal wall hernia for which reconstruction was needed and one patient had an intra-articular screw which had to be removed [16] (Table 5).

**Table 5:** Reasons for re-operation, by surgical approach

Study	SSI, *n* (%)	Implant related, *n* (%)	Neurological complications, *n* (%)	Non-union, *n* (%)	Additive fixation, *n* (%)	Hematoma, *n* (%)	Secondary placement THP, *n* (%)	Other, *n* (%)	Specification
**(Modified) Stoppa**									
Bastian et al. [16]	3 (50)	1^a^ (16.7)				1 (16.7)		1^b^ (16.7)	^a^Intra-articular screw ^b^Abdominal wall hernia
Isaacson et al. [30]	3 (100)								
Verbeek et al. [32]	2 (50)					1 (25)		1^a^ (25)	^a^Revision surgery due to loss of reduction
Overall, *n* (%)	13 (69.2)	1 (7.7)				2 (15.4)		1 (7.7)	
**Percutaneous approach**									
Chen et al. [18]			2 (100)						Screw replacement due to neurological complication
Dekimpe et al. [19]			2 (100)						Screw removal due to irritation
Falzerano et al		2 (100)							Screw removal due to mobilization of material
Li et al. [21]	1 (100)								
Osterhoff et al. [22]			2^a^ (33.3)	2 (33.4)	2 (33.3)				^a^Nerve irritation
Rommens et al. [23]	2 (25)	2^a^ (25)		1 (12.5)		1 (12.5)		2^b^ (25)	^a^Malposition of screws ^b^Infection and nonunion
Wenning et al. [24]	1 (16.7)	5^a^ (83.3)							^a^Mal position of screws
Overall, *n* (%)	4 (14.8)	9 (33.4)	6 (22.2)	3 (11.1)	2 (7.4)	1 (3.7)		2 (7.4)	
**External fixator**									
Scaglione et al. [25]	3 (100)								
Mean, *n* (%)	3 (100)								
**Kocher Langenbeck**									
Alexa et al. [33]	1 (50)						1 (50)		
Negrin et al. [35]							4 (100)		
Susuki et al. [36]	2 (100)								
Overall, *n* (%)	3 (37.5)						5 (62.5)		

*SSI* surgical site infections; *THP* total hip prosthesis

Four studies including 171 patients with an acetabular fracture reported on complications after ORIF using a (modified) Stoppa approach [29–32]. The overall complication rate was 9–25%. SSI rates were reported in all four studies (Table 2). One study reported an implant-related complication in one patient (2%), who needed revision surgery due to loss of reduction of the posterior column [32]. Neurological complications were present in up to 6% of patients [30, 31, 38]. Bastian et al. reported femoral palsy in one patient which resolved spontaneously within the follow-up period of 3 years [16]. Isaacson reported femoral cutaneous palsy in two patients that both needed a lateral window, one of which subsequently resolved spontaneously within the follow-up period [30]. Sing et al. reported persistent palsies up to 3–5 months of the obturator nerve and lateral cutaneous nerve in two (6%) patients [31]. One study reported non-union in 6% of patients [30]. Re-operations rates of 8% were reported in two studies [30, 32] (Table 2). The main reasons to perform re-operations were operative debridement because of SSI (*n = *5 patients), revision surgery because of loss of reduction (*n = *1 patient) and evacuation of post-operative hematoma (*n = *1 patient) (Table 5).

#### Percutaneous approach

A total of 390 patients suffering from a pelvic ring fracture were treated using a percutaneous approach, predominantly for screw fixation (Table 1). The overall complication rate for the percutaneous approach was 11.0% (Table 4). Five studies reported on the outcomes after sacroiliac screws [18, 20–22, 24], in one study a combination of sacroiliac screws with transpubic screws was used [19] and in one study only transpubic screws [23]. Two studies compared the results of the percutaneous approach with other surgical approaches [18, 24]. The study of Chen et al. compared the results after sacroiliac plate fixation (Group A) to sacroiliac screw fixation (Group B). In Group B two patients (7%) needed screw removal because of nerve compression leading to neurological pain (Table 5) [18]. Wenning et al. compared the results after sacroiliac screws fixations (Group A) with lumbo pelvic fixation (Group B) [24]. In Group A one patient (2%) suffered from deep infections and in five patients (11%) malposition of screws was found. All patients needed revision surgery (Table 5). The only RCT included in this review compared the results of percutaneous sacroiliac screw fixation with open anterior sacroiliac plate fixation. Post-operative infections were significantly more often seen in the control group (9%) compared to the percutaneous screw fixation group (3%) [21]. Falzarano et al. reported post-operative infections in 12% of the patients. None of the patients needed operative debridement and all infections were superficial, which were successfully treated using oral antibiotics [20]. Implant-related complications occurred most often in the study by Dekimpe et al. reporting these complications in 9% of the patients. Only one patient needed screw removal due to persistent irritation and psoas tendinitis because of a penetrating screw [19]. Re-operations were most often performed (24% of patients) in the study by Osterhoff et al. [22]. Overall for the percutaneous approach, the main reasons to perform re-operation were implanted-related (33.4%) (Table 5).

#### External fixator placement

Four studies with a total of 116 pelvic ring fracture patients reported the outcomes after treatment with external fixator used as definitive fixation [25–28]. The overall complication rate for the external fixator placement was 31% predominantly caused by SSI due to pin tract infections (22%) and implant-related complications 13% such as malpositioning of the screws of the external fixator (Table 4). Three studies included high-energy trauma patients and one study included low-energy trauma patients. The study by Scaglione et al. included 37 patients receiving an external fixator as definitive treatment. In four patients, external fixation was followed by definitive internal fixation [25].

The study by Bi et al. compared the results after anterior external fixation (Group A) to modified pedicle rods (Group B) [26]. Pin tract infections were observed for six (27%) patients in Group A compared to 0% in Group B. Implant-related complications were reported for five (23%) patients in Group A and included loosening of implants with consecutive loss of fixation. Neurological complications occurred in two (9%) of the patients treated with external fixation. All neurological complications comprised of temporary lateral femoral cutaneous nerve (LFCN) palsy and resolved spontaneously without residual symptoms. The infection rate reported by Bi et al. for the group treated with an external fixator (Group A: 27%) was the second highest reported by the studies included in this review [26]. Scaglione et al. reported even more post-operative infections, all pin tract infections, in 35% of the included patients [25]. However, in most of the cases the infection was superficial and successfully treated with oral antibiotics. Only in three patients, removal of pins was necessary. Neurological complications were reported to be absent in two studies [25, 27]. The remaining studies reported neurological complications in 9% and 10% [26, 28]. In the study by Bi et al. two (9%) patients suffered from lateral femoral nerve palsy after external fixation and Vécsei et al. reported no further specified nerve lesions in two (10%) of their patients [26, 28]. Noticeably, the study population included by Vécsei comprised patients with severe pelvic ring fractures and significant associated injuries. Eight patients (28.6%) died upon arrival at the hospital [28].

#### Kocher–Langenbeck approach

Five studies included 396 patients who underwent surgery after an acetabulum fracture via a Kocher–Langenbeck approach [33–37]. The overall complication rate ranged from 3 to 12%. For this approach, the most frequently encountered complications were neurological, documented in 2% [37] 7% [33] and 12% [35] of patients (Table 4). Three patients in the study by Alexa et al. suffered from peroneal-nerve palsies which resolved within three months after trauma [33]. Negrin et al. reported neurological palsies in two (12%) patients, one of which suffered from persistent weakness in sensation and Kumar et al. reported sciatic nerve palsy in two patients. Both studies did not provide further details [35, 37]. Non-union was not reported in any of the patients in two studies [34, 36]. Re-operation rates ranged between 5 and 15% [33, 35, 36] and involved operative debridement due to SSI in three patients (37.5%) and secondary placement of a total hip prosthesis in five patients (62.5%) (Table 5).

#### Ilioinguinal approach

One study reported SSI in 7% of the patients treated with a reconstruction plate via an ilioinguinal approach for their acetabular fractures [34]. Other post-operative complications were not reported (Table 2).

### Reported risk factors for post-operative complications

Possible risk factors for post-operative SSI were identified by two of the included studies [34, 36]. Both studies reported the outcomes after surgical fixation of patients suffering from acetabular fractures. Iqbal et al. found that concomitant abdominal injuries, (odds ratio [OR] 19.3; 95% confidence interval [CI] 0.83–1.32; *p = *0.002), prolonged ICU stay (OR 18.3; 95% CI 0.88–1.22; *p = *0.002), body mass index (OR: 14.2; 95% CI 0.91–1.32; *p = *0.003) and prolonged operation time (OR 9.50; 95% CI 1.12–1.56; *p = *0.008) were associated with increased risk for SSI [34]. Suzuki et al. also identified body mass index and ICU stay as statistically significant risk factors for post-operative SSI after acetabular fracture surgery in a univariable analysis [36].

## Discussion

This systematic review provides a comprehensive overview of the literature on post-operative complications for surgical approaches that are used in acetabular and pelvic ring fracture surgery. Whenever possible, we also documented the identified risk factors for post-operative complications.

### Surgical approaches for pelvic ring fractures

Comparing the overall complications rates between the included approaches the highest overall complication percentage (31.1%) was reported in patients treated with an external fixator as definitive fixation. Most of the complications observed in these groups were SSI (22.1%) and concerned pin tract infections (Table 4). The study by Scaglione et al. reported the highest number with SSI percentage of 34%. However, it is important to notice that in most of the cases these SSIs were superficial and could successfully be treated with oral antibiotics. Deep infection subsequently needing removal of the external fixator pins was only necessary in three patients and no cases of osteomyelitis were reported [25] (Table 5). In patients with complex unstable pelvic ring fractures and signs of hemodynamic instability after high-energy trauma, temporary emergency stabilisation using an external fixator is inevitable for obtaining early stabilisation of both patient and fracture [39]. However, as also illustrated in this review, external fixators are notorious for high infection rates, which is explained by the persistent port d’entree caused by the external fixator pins penetrating the skin. Still in many cases, the infections are limited to the superficial subcutaneous tissue and deep infections including osteomyelitis are rare. However, adequate pin tract hygiene, frequent inspection and reducing the period to a minimum between the emergency and definite fixation is essential to reduce risks of SSI and help in early recognition preventing deterioration to deep infections.

The lowest SSI rates were reported for the Kocher–Langenbeck approach (3.1%) and the percutaneous approach (4.4%) (Table 4). Minimally invasive surgery, using smaller incisions and percutaneous insertions of screws, inflicts less tissue damage and minimal wound exposure during surgery, leading to lower post-operative SSI rates. However, especially in complex acetabular and pelvic ring fractures, sufficient exposure may be needed to achieve adequate restoration of the joint surface and fixation of the fracture. Since minimally invasive and percutaneous techniques provide limited exposure and visualisation of the fracture, the risks of these techniques may include imperfect fracture reduction and fixation [40].

The modified Stoppa approach is currently widely used for pelvic ring and acetabular fractures and was introduced to avoid dissection of the inguinal canal, femoral artery, and external iliac vessel. This minimized the risk of iatrogenic damage to these structures while still providing adequate fracture exposure [41]. This assumption is substantiated by the finding of the current review. For the Kocher–Langenbeck approach, a higher overall neurological complication rate of 5.2% was reported compared to the Modified Stoppa approach (1.1%). Sciatic nerve damage after acetabular fractures may result from the injury itself or from iatrogenic intra-operative neurological damage during especially deep dissection when using the Kocher–Langenbeck approach [42]. The risk of damaging the sciatic nerve during surgery can be reduced by clear identification and tracing the nerve prior to the division of the external rotator muscles. However, it is important to understand that extensive dissection for identification purposes can skeletonize the sciatic nerve and thus damage its blood supply [11].

The reasons for performing re-operations differed between the included surgical approaches. For the modified Stoppa approach and external fixation, the most prevalent reason for re-operations including operative debridement was SSI, in 69% and 100% of cases, respectively. In the studies reporting on percutaneous approach hardware removal or additional fixation due to implant related complications (malposition or loss of fixation) were the main reasons (33.4%) for re-operations (Table 4). The extent of re-operations due to SSI differed among the included studies from simple debridement followed by a short period of oral antibiotics to removal of fixation material with extended periods of intravenous antibiotics. Earlier published studies demonstrated that re-operations in trauma patients are one of the main causes of long-term reduced functional outcomes [43, 44]. It may be assumed that the same is true for the group of patients with pelvic ring and/or acetabular fractures. The available and included studies do unfortunately not allow for a quantitative substantiation of this assumption. The current literature is heterogeneous with respect to fracture characteristics, reported functionality outcomes and follow-up periods.

### Associated risk factors

Reports on potentially associated risk factors for post-operative complications in pelvic fracture patients are scarce in the currently available literature. In this review, only two studies identified the following risk factors associated with one specific post-operative complication, i.e., SSI; concomitant abdominal injuries, body mass index (BMI), prolonged ICU stay and operation time [34, 36]. The presence of concomitant (abdominal) traumatic injuries may induce extensive traumatic tissue damage, resulting in increased (internal) wound surfaces, possible port d’entrée and hematoma’s subsequently attributing to impaired wound healing [34]. A high BMI was also found to be a significant risk factor for developing post-operative infections. In general, obese patients have an increased risk of (peri)operative complications induced by anaesthesia and surgery [45]. Wound healing problems were specifically seen in obese patients. Several underlying mechanisms such as decreased tissue oxygenation, impaired inflammatory response and malnutrition contribute to this increased risk of wound infections [46].

### Limitations

Although post-operative complications after pelvic fractures are frequently addressed in the current literature, many of the available studies are small, have a retrospective study design and a substantial risk of bias. Only one small study was found that reported complications after the ilioinguinal approach. Large and well-designed comparative prospective studies and randomized controlled trials are still lacking. Furthermore, only two studies in this review reported on potential risk factors for development of post-operative SSI’s and no studies reporting on risk factors for other post-operative complications were found.

## Conclusion

Complications after commonly used surgical approaches for fixation of pelvic ring and acetabular fractures are frequently reported with overall complications rates up to 31%. External fixation of the pelvic ring is associated with the highest numbers of complications including mainly SSI’s and neurological complications. Studies identifying potential risk factors for post-operative complications are scarce. More research is needed for a better understanding of risk factors for post-operative complications after different surgical approaches for pelvic ring and acetabular fractures. Enhanced insight in this matter can help surgeons to better understand the risks their patients are exposed to and assist in development of preventive strategies and (pre)operative decision making.

## Supplementary Information

Below is the link to the electronic supplementary material.

Supplementary file1 (DOCX 15 kb)

## References

[1] Giannoudis PV, Grotz MR, Tzioupis C, Dinopoulos H, Wells GE, Bouamra O (2007). Prevalence of pelvic fractures, associated injuries, and mortality: the United Kingdom perspective. J Trauma.

[2] Schmal H, Markmiller M, Mehlhorn AT, Sudkamp NP (2005). Epidemiology and outcome of complex pelvic injury. Acta Orthop Belg.

[3] Buller LT, Best MJ, Quinnan SM (2016). A nationwide analysis of pelvic ring fractures: incidence and trends in treatment, length of stay, and mortality. Geriatr Orthop Surg Rehabil.

[4] Pereira GJC, Damasceno ER, Dinhane DI, Bueno FM, Leite JBR, Ancheschi BDC (2017). Epidemiology of pelvic ring fractures and injuries. Rev Bras Ortop.

[5] Vaidya R, Scott AN, Tonnos F, Hudson I, Martin AJ, Sethi A (2016). Patients with pelvic fractures from blunt trauma. What is the cause of mortality and when?. Am J Surg.

[6] Yoshihara H, Yoneoka D (2014). Demographic epidemiology of unstable pelvic fracture in the United States from 2000 to 2009: trends and in-hospital mortality. J Trauma Acute Care Surg.

[7] Costantini TW, Coimbra R, Holcomb JB, Podbielski JM, Catalano RD, Blackburn A (2017). Pelvic fracture pattern predicts the need for hemorrhage control intervention-results of an AAST multi-institutional study. J Trauma Acute Care Surg.

[8] Bible JE, Wegner A, McClure DJ, Kadakia RJ, Richards JE, Bauer JM (2014). One-year mortality after acetabular fractures in elderly patients presenting to a level-1 trauma center. J Orthop Trauma.

[9] Wollmerstädt J, Pieroh P, Schneider I, Zeidler S, Höch A, Josten C (2020). Mortality, complications and long-term functional outcome in elderly patients with fragility fractures of the acetabulum. BMC Geriatr.

[10] Grubor P, Milicevic S, Biscevic M, Tanjga R (2011). Selection of treatment method for pelvic ring fractures. Med Arh.

[11] Küper MA, Trulson A, Minarski C, Stuby F, Stöckle U, Konrads C (2021). Risks and strategies to avoid approach-related complications during operative treatment of pelvic ring or acetabular fractures. Z Orthop Unfall.

[12] Becker SC, Holstein JH, Pizanis A, Pohlemann T (2013). Anterior approaches to the pelvic ring. Unfallchirurg.

[13] Ziran N, Soles GLS, Matta JM (2019). Outcomes after surgical treatment of acetabular fractures: a review. Patient Saf Surg.

[14] Liberati A, Altman DG, Tetzlaff J, Mulrow C, Gotzsche PC, Ioannidis JP (2009). The PRISMA statement for reporting systematic reviews and meta-analyses of studies that evaluate healthcare interventions: explanation and elaboration. BMJ (Clinical research ed).

[15] Slim K, Nini E, Forestier D, Kwiatkowski F, Panis Y, Chipponi J (2003). Methodological index for non-randomized studies (minors): development and validation of a new instrument. ANZ J Surg.

[16] Bastian JD, Ansorge A, Tomagra S, Siebenrock KA, Benneker LM, Büchler L (2016). Anterior fixation of unstable pelvic ring fractures using the modified Stoppa approach: mid-term results are independent on patients' age. Eur J Trauma Emerg Surg.

[17] Oh HK, Choo SK, Kim JJ, Lee M (2016). Stoppa approach for anterior plate fixation in unstable pelvic ring injury. Clin Orthop Surg.

[18] Chen HW, Liu GD, Fei J, Yi XH, Pan J, Ou S (2012). Treatment of unstable posterior pelvic ring fracture with percutaneous reconstruction plate and percutaneous sacroiliac screws: a comparative study. J Orthop Sci.

[19] Dekimpe C, Andreani O, De Dompsure RB, Lemmex DB, Layet V, Foti P (2020). CT-guided fixation of pelvic fractures after high-energy trauma, by interventional radiologists: technical and clinical outcome. Eur Radiol.

[20] Falzarano G, Rollo G, Bisaccia M, Pace V, Lanzetti RM, Garcia-Prieto E (2018). Percutaneous screws CT guided to fix sacroiliac joint in tile C pelvic injury. Outcomes at 5 years of follow-up. Sicot J.

[21] Li CL (2014). Clinical comparative analysis on unstable pelvic fractures in the treatment with percutaneous sacroiliac screws and sacroiliac joint anterior plate fixation. Eur Rev Med Pharmacol Sci.

[22] Osterhoff G, Ossendorf C, Wanner GA, Simmen HP, Werner CM (2011). Posterior screw fixation in rotationally unstable pelvic ring injuries. Injury.

[23] Rommens PM, Graafen M, Arand C, Mehling I, Hofmann A, Wagner D (2020). Minimal-invasive stabilization of anterior pelvic ring fractures with retrograde transpubic screws. Injury.

[24] Wenning KE, Yilmaz E, Schildhauer TA, Hoffmann MF (2021). Comparison of lumbopelvic fixation and iliosacral screw fixation for the treatment of bilateral sacral fractures. J Orthop Surg Res.

[25] Scaglione M, Parchi P, Digrandi G, Latessa M, Guido G (2010). External fixation in pelvic fractures. Musculoskelet Surg.

[26] Bi C, Wang Q, Wu J, Zhou F, Zhang F, Liang H (2017). Modified pedicle screw-rod fixation versus anterior pelvic external fixation for the management of anterior pelvic ring fractures: a comparative study. J Orthop Surg Res.

[27] Gänsslen A, Hildebrand F, Kretek C (2013). Supraacetabular external fixation for pain control in geriatric type B pelvic injuries. Acta Chir Orthop Traumatol Cech.

[28] Vécsei V, Negrin LL, Hajdu S (2010). Today's role of external fixation in unstable and complex pelvic fractures. Eur J Trauma Emerg Surg.

[29] Khoury A, Weill Y, Mosheiff R (2012). The Stoppa approach for acetabular fracture. Oper Orthop Traumatol.

[30] Isaacson MJ, Taylor BC, French BG, Poka A (2014). Treatment of acetabulum fractures through the modified Stoppa approach: strategies and outcomes. Clin Orthop Relat Res.

[31] Singh SV, Chopra RK, Puri G, Pheroz M, Kumar S, Bansal A (2020). Clinico-radiological evaluation of modified stoppa approach in treatment of acetabulum fractures. Cureus.

[32] Verbeek DO, Ponsen KJ, van Heijl M, Goslings JC (2018). Modified Stoppa approach for operative treatment of acetabular fractures: 10-year experience and mid-term follow-up. Injury.

[33] Alexa O, Malancea RI, Puha B, Luncă S, Veliceasa B (2013). Results of surgical treatment of acetabular fractures using Kocher-Langenbeck approach. Chirurgia (Bucur).

[34] Iqbal F, Younus S, Asmatullah, Zia OB, Khan N (2017). Surgical site infection following fixation of acetabular fractures. Hip Pelvis.

[35] Negrin LL, Seligson D (2010). The Kocher-Langenbeck approach: differences in outcome of transverse acetabular fractures depending on the patient's position. Eur J Trauma Emerg Surg.

[36] Suzuki T, Morgan SJ, Smith WR, Stahel PF, Gillani SA, Hak DJ (2010). Postoperative surgical site infection following acetabular fracture fixation. Injury.

[37] Kumar D, Kushwaha NS, Tiwari PG, Sharma Y, Srivastava RN, Sharma V (2021). Outcome of acetabulum fractures treated with open reduction and internal fixation through Kocher-Langenbeck approach: a retrospective study. J Clin Orthop Trauma.

[38] Khoury A, Weill Y, Mosheiff R (2012). The Stoppa approach for acetabular fracture Twijfel: verlengde stoppa, wel inclusie?. Oper Orthop Traumatol.

[39] Skitch S, Engels PT (2018). Acute management of the traumatically injured pelvis. Emerg Med Clin N Am.

[40] Elzohairy MM, Salama AM (2017). Open reduction internal fixation versus percutaneous iliosacral screw fixation for unstable posterior pelvic ring disruptions. Orthop Traumatol Surg Res.

[41] Stoppa RE (1989). The treatment of complicated groin and incisional hernias. World J Surg.

[42] Issack PS, Helfet DL (2009). Sciatic nerve injury associated with acetabular fractures. Hss j.

[43] Fang C, Wong TM, To KK, Wong SS, Lau TW, Leung F (2017). Infection after fracture osteosynthesis-part II. J Orthop Surg (Hong Kong).

[44] Lee JM, Herrera-Escobar J, Apoj M, Al Rafai SS, Han K, Nehra D (2019). The impact of in-hospital complications on the long-term functional outcome of trauma patients: a multicenter study. Surgery.

[45] Bazurro S, Ball L, Pelosi P (2018). Perioperative management of obese patient. Curr Opin Crit Care.

[46] Pierpont YN, Dinh TP, Salas RE, Johnson EL, Wright TG, Robson MC (2014). Obesity and surgical wound healing: a current review. ISRN Obes.

