# Supplementary material for: The relation between surgical approaches for pelvic ring and acetabular fractures and postoperative complications: a systematic review
Source: Eur J Trauma Emerg Surg. 2022 Nov 25;49(2):709–22. doi: 10.1007/s00068-022-02118-3 (PMC10175345; doi:10.1007/s00068-022-02118-3)
Supplement: Supplementary file 1 — Supplementary file1 (DOCX 15 kb) [file 68_2022_2118_MOESM1_ESM.docx]

**Appendix I.** Search strategies

**Pubmed search term:**

(("pelvic ring fractures"[tw] OR "pelvic ring fracture"[tw] OR "acetabular fractures"[tw] OR "acetabular fracture"[tw] OR "acetabulum fractures"[tw] OR "acetabulum fracture"[tw] OR "pelvic fractures"[tw] OR "pelvic fracture"[tw] OR "pelvis fractures"[tw] OR "pelvis fracture"[tw] OR (("Fractures, Bone"[majr] OR "fracture"[ti] OR "fractures"[ti] OR "fractured"[ti] OR "fracturing"[ti] OR "fractur*"[ti]) AND ("Acetabulum"[majr] OR "Pelvic Ring"[ti] OR "Pelvic Rings"[ti] OR "Pelvis Ring"[ti] OR "Acetabulum"[ti] OR "Acetabular"[ti] OR "Acetabul*"[ti])) OR ("Fractures, Bone"[majr] AND ("Pelvic Bones"[majr] OR "Pelvis"[majr] OR "Acetabulum"[majr])) OR (("Fractures, Bone"[majr] OR "fracture"[ti] OR "fractures"[ti] OR "fractured"[ti] OR "fracturing"[ti] OR "fractur*"[ti]) AND ("Pelvic Bones"[majr] OR "Pelvis"[majr] OR "Acetabulum"[majr] OR "Pelvic Ring"[ti] OR "Pelvic Rings"[ti] OR "Pelvis Ring"[ti] OR "Pelvic"[ti] OR "Pelvis"[ti] OR "pelvi*"[ti] OR "Acetabulum"[ti] OR "Acetabular"[ti] OR "Acetabul*"[ti]))) AND ("Young and Burgess"[tw] OR "young burgess"[tw] OR ("Young"[tw] AND "Burgess"[tw]) OR "Tile's classification"[tw] OR "Tile classification"[tw] OR "Tile"[tw] OR "Rommens"[tw] OR "letournel"[tw] OR "Trauma mechanism"[tw] OR "Trauma mechanisms"[tw] OR "High Energy"[tw] OR "Accidents"[Mesh] OR "Accidental Falls"[Mesh] OR "Fall"[tw] OR "falls"[tw] OR "falling"[tw] OR "accidents"[tw] OR "accident"[tw] OR "crash"[tw] OR "crashes"[tw] OR "crashed"[tw] OR "collision"[tw] OR "collisions"[tw] OR "collisioned"[tw] OR "accidental"[tw] OR "trauma"[ti] OR "traumas"[ti] OR "traumatic"[ti] OR "Risk Factors"[mesh] OR "risk factor"[ti] OR "risk factors"[ti]) AND ("Postoperative Complications"[majr] OR "Pain, Postoperative"[majr] OR "Postoperative Cognitive Complications"[majr] OR "Postoperative Hemorrhage"[majr] OR "Prosthesis-Related Infections"[majr] OR "Surgical Wound Dehiscence"[majr] OR "Surgical Wound Infection"[majr] OR "postoperative complications"[tiab] OR "postoperative complication"[tiab] OR "post operative complications"[tiab] OR "post operative complication"[tiab] OR "peroperative complications"[tiab] OR "peroperative complication"[tiab] OR "per operative complications"[tiab] OR "per operative complication"[tiab] OR "perioperiative complications"[tiab] OR "perioperiative complication"[tiab] OR "peri operiative complications"[tiab] OR "peri operiative complication"[tiab] OR "Intraoperative Complications"[majr] OR "Intraoperative Complications"[tiab] OR "Intraoperative Complication"[tiab] OR "Intra operative Complications"[tiab] OR "Intra operative Complication"[tiab] OR "medical complications"[tiab] OR "medical complication"[tiab] OR "wound complications"[tiab] OR "wound complication"[tiab] OR "Wound Infection"[majr] OR "Wound Infections"[tiab] OR "Wound Infection"[tiab] OR "leak"[tiab] OR "leaks"[tiab] OR "leaking"[tiab] OR "Hemorrhage"[majr:NoExp] OR "haemorrhages"[tiab] OR "haemorrhage"[tiab] OR "haemorrhaging"[tiab] OR "hemorrhages"[tiab] OR "hemorrhage"[tiab] OR "haemorrhaging"[tiab] OR "bleeding"[tiab] OR "bleed"[tiab] OR "bleeds"[tiab] OR "Hematoma"[majr] OR "Hematoma"[tiab] OR "Hematoma"[tiab] OR "Haematoma"[tiab] OR "Haematoma"[tiab] OR "material complications"[tiab] OR "material complication"[tiab] OR "Surgical Equipment/adverse effects"[majr] OR "outbreak of material"[tiab] OR "neurogenic complications"[tiab] OR "neurogenic complication"[tiab] OR "Chronic Pain"[majr] OR "Chronic Pain"[tiab] OR "Chronic Pains"[tiab] OR "nerve damage"[tiab] OR "nerve injuries"[tiab] OR "nerve injury"[tiab] OR "Peripheral Nerves/injuries"[majr] OR "Nervous System/injuries"[majr] OR "Neurological damage"[tiab] OR "Neurologic damage"[tiab] OR "Neurological injuries"[tiab] OR "Neurologic injuries"[tiab] OR "Neurological injury"[tiab] OR "Neurologic injury"[tiab] OR "Reoperation"[majr] OR "Reoperation"[tiab] OR "Reoperations"[tiab] OR "Re operation"[tiab] OR "Re operations"[tiab] OR "Repeat Surgery"[tiab] OR "Revision Surgery"[tiab] OR "Hospital Mortality"[majr] OR "Hospital Mortality"[tiab] OR "Inhospital Mortality"[tiab] OR "Mortality"[majr] OR "Mortality"[tiab] OR "deaths"[tiab] OR "death rate"[tiab] OR "death rates"[tiab] OR "secondary total hip"[tiab] OR "secondary hip"[tiab] OR "secondary tha"[tiab] OR "secondary thr"[tiab] OR "Fractures, Bone/mortality"[majr] OR "infection"[tiab] OR "infections"[tiab] OR "infected"[tiab] OR "Surgical Procedures, Operative/complications"[majr] OR "complications"[ti] OR "complication"[ti]) NOT (("Infant"[mesh] OR "Child"[mesh] OR "Adolescent"[mesh] OR "Infant"[ti] OR "Child"[ti] OR "Adolescent"[ti] OR "Infants"[ti] OR "Children"[ti] OR "Adolescents"[ti] OR "Infancy"[ti] OR "Childhood"[ti] OR "Adolescence"[ti] OR "pediatric"[ti] OR "paediatric"[ti] OR "pediatrics"[ti] OR "paediatrics"[ti]) NOT ("Adult"[mesh] OR "adult"[ti] OR "adults"[ti] OR "elderly"[ti])) AND ("1990/01/01"[PDAT] : "3000/12/31"[PDAT]) AND ("English"[la] OR "Dutch"[la] OR "German"[la]) NOT (("Case Reports"[ptyp] OR "case report"[ti]) NOT ("Review"[ptyp] OR "review"[ti] OR "Clinical Study"[ptyp] OR "trial"[ti] OR "RCT"[ti])))

**Embase search term:**

(("pelvic ring fractures".ti,ab OR "pelvic ring fracture".ti,ab OR "acetabular fractures".ti,ab OR "acetabular fracture".ti,ab OR "acetabulum fractures".ti,ab OR "acetabulum fracture".ti,ab OR exp *" pelvis fracture"/ OR "pelvic fractures".ti,ab OR "pelvic fracture".ti,ab OR "pelvis fractures".ti,ab OR "pelvis fracture".ti,ab OR ((exp *"Fracture"/ OR "fracture".ti OR "fractures".ti OR "fractured".ti OR "fracturing".ti OR "fractur*".ti) AND (*"Acetabulum"/ OR "Pelvic Ring".ti OR "Pelvic Rings".ti OR "Pelvis Ring".ti OR "Acetabulum".ti OR "Acetabular".ti OR "Acetabul*".ti)) OR (exp *"Fracture"/ AND (exp *"Pelvic Girdle"/ OR *"Pelvis"/ OR exp *"Acetabulum"/)) OR ((exp *"Fracture"/ OR "fracture".ti OR "fractures".ti OR "fractured".ti OR "fracturing".ti OR "fractur*".ti) AND (exp *"Pelvic Girdle"/ OR *"Pelvis"/ OR exp *"Acetabulum"/ OR "Pelvic Ring".ti OR "Pelvic Rings".ti OR "Pelvis Ring".ti OR "Pelvic".ti OR "Pelvis".ti OR "pelvi*".ti OR "Acetabulum".ti OR "Acetabular".ti OR "Acetabul*".ti))) AND ("Young and Burgess".ti,ab OR "young burgess".ti,ab OR ("Young".ti,ab AND "Burgess".ti,ab) OR "Tile's classification".ti,ab OR "Tile classification".ti,ab OR "Tile".ti,ab OR "Rommens".ti,ab OR "letournel".ti,ab OR "Trauma mechanism".ti,ab OR "Trauma mechanisms".ti,ab OR "High Energy".ti,ab OR exp *"Accident"/ OR *"Falling"/ OR "Fall".ti,ab OR "falls".ti,ab OR "falling".ti,ab OR "accidents".ti,ab OR "accident".ti,ab OR "crash".ti,ab OR "crashes".ti,ab OR "crashed".ti,ab OR "collision".ti,ab OR "collisions".ti,ab OR "collisioned".ti,ab OR "accidental".ti,ab OR "trauma".ti OR "traumas".ti OR "traumatic".ti OR "trauma".af OR "traumas".af OR "traumatic".af OR "traumatology".af) AND (exp *"Fracture Fixation"/ OR "Fracture Fixation".ti OR "osteosynthesis".ti OR "Fixation".ti) AND ("Postoperative".ti,ab OR "Postoperativ*".ti,ab OR exp *"Postoperative Complication"/ OR *"Wound Dehiscence"/ OR "postoperative complications".ti,ab OR "postoperative complication".ti,ab OR "post operative complications".ti,ab OR "post operative complication".ti,ab OR "peroperative complications".ti,ab OR "peroperative complication".ti,ab OR "per operative complications".ti,ab OR "per operative complication".ti,ab OR "perioperiative complications".ti,ab OR "perioperiative complication".ti,ab OR "peri operiative complications".ti,ab OR "peri operiative complication".ti,ab OR "Intraoperative Complications"/ OR "Intraoperative Complications".ti,ab OR "Intraoperative Complication".ti,ab OR "Intra operative Complications".ti,ab OR "Intra operative Complication".ti,ab OR "medical complications".ti,ab OR "medical complication".ti,ab OR "wound complications".ti,ab OR "wound complication".ti,ab OR *"Wound Infection"/ OR "Wound Infections".ti,ab OR "Wound Infection".ti,ab OR "leak".ti,ab OR "leaks".ti,ab OR "leaking".ti,ab OR *"Bleeding"/ OR "haemorrhages".ti,ab OR "haemorrhage".ti,ab OR "haemorrhaging".ti,ab OR "hemorrhages".ti,ab OR "hemorrhage".ti,ab OR "haemorrhaging".ti,ab OR "bleeding".ti,ab OR "bleed".ti,ab OR "bleeds".ti,ab OR *"Hematoma"/ OR "Hematoma".ti,ab OR "Hematoma".ti,ab OR "Haematoma".ti,ab OR "Haematoma".ti,ab OR "material complications".ti,ab OR "material complication".ti,ab OR exp *"surgical equipment"/am OR "outbreak of material".ti,ab OR "neurogenic complications".ti,ab OR "neurogenic complication".ti,ab OR *"Chronic Pain"/ OR "Chronic Pain".ti,ab OR "Chronic Pains".ti,ab OR "nerve damage".ti,ab OR "nerve injuries".ti,ab OR "nerve injury".ti,ab OR exp *"Nerve injury"/ OR "Neurological damage".ti,ab OR "Neurologic damage".ti,ab OR "Neurological injuries".ti,ab OR "Neurologic injuries".ti,ab OR "Neurological injury".ti,ab OR "Neurologic injury".ti,ab OR *"Reoperation"/ OR "Reoperation".ti,ab OR "Reoperations".ti,ab OR "Re operation".ti,ab OR "Re operations".ti,ab OR "Repeat Surgery".ti,ab OR "Revision Surgery".ti,ab OR *"Hospital Mortality"/ OR "Hospital Mortality".ti,ab OR "Inhospital Mortality".ti,ab OR exp *"Mortality"/ OR "Mortality".ti,ab OR "deaths".ti,ab OR "death rate".ti,ab OR "death rates".ti,ab OR "secondary total hip".ti,ab OR "secondary hip".ti,ab OR "secondary tha".ti,ab OR "secondary thr".ti,ab OR "infection".ti,ab OR "infections".ti,ab OR "infected".ti,ab OR "complications".ti OR "complication".ti) NOT ((exp "Infant"/ OR exp "Child"/ OR exp "Adolescent"/ OR "Infant".ti OR "Child".ti OR "Adolescent".ti OR "Infants".ti OR "Children".ti OR "Adolescents".ti OR "Infancy".ti OR "Childhood".ti OR "Adolescence".ti OR "pediatric".ti OR "paediatric".ti OR "pediatrics".ti OR "paediatrics".ti) NOT (exp "Adult"/ OR "adult".ti OR "adults".ti OR "elderly".ti)) AND (1990 OR 1991 OR 1992 OR 1993 OR 1994 OR 1995 OR 1996 OR 1997 OR 1998 OR 1999 OR 2000 OR 2001 OR 2002 OR 2003 OR 2004 OR 2005 OR 2006 OR 2007 OR 2008 OR 2009 OR 2010 OR 2011 OR 2012 OR 2013 OR 2014 OR 2015 OR 2016 OR 2017 OR 2018 OR 2019 OR 2020 OR 2021 OR 2022).yr AND ("English".la OR "Dutch".la OR "German".la) NOT (("Case Report"/ OR "case report".ti) NOT (exp "Review"/ OR "review".ti OR "Clinical Study"/ OR exp "Clinical Trial"/ OR "trial".ti OR "RCT".ti)) NOT (conference review or conference abstract).pt)

Results 12^th^ of February 2022: 1396 articles after removal of duplicates
